# Association of Thymidylate Synthase Polymorphisms with Acute Pancreatitis and/or Peripheral Neuropathy in HIV-Infected Patients on Stavudine-Based Therapy

**DOI:** 10.1371/journal.pone.0057347

**Published:** 2013-02-28

**Authors:** Pere Domingo, Maria del Carmen Cabeza, Ferran Torres, Juliana Salazar, Maria del Mar Gutierrez, Maria Gracia Mateo, Esteban Martínez, Joan Carles Domingo, Irene Fernandez, Francesc Villarroya, Esteban Ribera, Francesc Vidal, Montserrat Baiget

**Affiliations:** 1 Infectious Diseases Unit, Hospital de la Santa Creu i Sant Pau, Universitat Autònoma de Barcelona, Barcelona, Spain; 2 Biostatistics and Data Management Platform, IDIBAPS, Hospital Clinic; Biostatistics Unit, School of Medicine, Universitat Autònoma de Barcelona, Barcelona, Spain; 3 CIBERER (U-705), Barcelona, Spain; 4 Infectious Diseases Service, Hospital Clínic Universitari, Universitat de Barcelona, Barcelona, Spain; 5 Department of Biochemistry and Molecular Biology, University of Barcelona, Barcelona, Spain; 6 Infectious Diseases Service, Hospital Vall d’Hebron, Universitat Autònoma de Barcelona, Barcelona, Spain; 7 Hospital Universitari Joan XXIII, Universitat Rovira I Virgili, Tarragona, Spain; 8 Department of Genetics, Hospital de la Santa Creu i Sant Pau, Universitat Autònoma de Barcelona, Barcelona, Spain; IPO, Inst Port Oncology, Portugal

## Abstract

**Background:**

Low expression thymidylate synthase (TS) polymorphism has been associated with increased stavudine triphosphate intracellular (d4T-TP) levels and the lipodystrophy syndrome. The use of d4T has been associated with acute pancreatitis and peripheral neuropathy. However, no relationship has ever been proved between TS polymorphisms and pancreatitis and/or peripheral neuropathy.

**Methods:**

We performed a case-control study to assess the relationship of TS and methylene-tetrahydrofolate reductase (MTHFR) gene polymorphisms with acute pancreatitis and/or peripheral neuropathy in patients exposed to d4T. Student’s t test, Pearson’s correlations, one-way ANOVA with Bonferroni correction and stepwise logistic regression analyses were done.

**Results:**

Forty-three cases and 129 controls were studied. Eight patients (18.6%) had acute pancreatitis, and 35 (81.4%) had peripheral neuropathy. Prior AIDS was more frequent in cases than in controls (OR = 2.36; 95%CI 1.10–5.07, P = 0.0247). L7ow expression TS and MTHFR genotype associated with increased activity were more frequent in patients with acute pancreatitis and/or peripheral neuropathy than in controls (72.1% vs. 46.5%, OR = 2.97; 95%CI: 1.33–6.90, P = 0.0062, and 79.1% vs. 56.6%, OR = 2.90, 95%CI: 1.23–7.41, P = 0.0142, respectively). Independent positive or negative predictors for the development of d4T-associated pancreatitis and/or peripheral neuropathy were: combined TS and MTHFR genotypes (reference: A+A; P = 0.002; OR_A+B_ = 0.34 [95%CI: 0.08 to 1.44], OR_B+A_ = 3.38 [95%CI: 1.33 to 8.57], OR_B+B_ = 1.13 [95%CI: 0.34 to 3.71]), nadir CD4 cell count >200 cells/mm^3^ (OR = 0.38; 95%CI: 0.17–0.86, P = 0.021), and HALS (OR = 0.39 95%CI: 0.18–0.85, P = 0.018).

**Conclusions:**

Low expression TS plus a MTHFR genotype associated with increased activity is associated with the development of peripheral neuropathy in d4T-exposed patients.

## Introduction

The doubtless efficacy of highly active antiretroviral therapy (HAART) is still shadowed by drug toxicity, specially that appearing in the long-term [1]. Some of the most important toxic effects experienced by patients on HAART are caused by the nucleoside reverse transcriptase inhibitor (NRTI) part of the regime [2]. Among NRTI-associated toxicity, that caused by thymidine analogues, namely zidovudine (AZT) and stavudine (d4T) stands out. The common mechanism underlying thymidine analogues’ toxic effects is mitochondrial toxicity [3]. The ability of the triphosphate active NRTI to inhibit mitochondrial gamma polymerase causes mitochondrial DNA depletion that may eventually lead to mitochondrial respiratory chain protein depletion which in turn leads to mitochondrial dysfunction [4]. This can ultimately cause cellular dysfunction or even cellular death [5].

The ability of thymidine analogues to inhibit mitochondrial gamma polymerase will depend on the intracellular concentrations of their triphosphate metabolites [6]. Therefore, all the circumstances increasing the levels of these metabolites may be accompanied by a higher degree of mitochondrial toxicity [7]. We recently described how thymidylate synthase (TS) polymorphisms may modify d4T triphosphate (d4T-TP) intracellular concentrations, and how higher intracellular levels of d4T-TP are associated with the HIV/HAART-associated lypodystrophy syndrome (HALS) [7], [8].

Our working hypothesis was that TS and MTHFR polymorphisms could be associated with other manifestations of mitochondrial toxicity, such as d4T-associated pancreatitis and d4T-associated peripheral neuropathy. To test this hypothesis we performed a case-control study in d4T-exposed patients who developed pancreatitis or peripheral neuropathy while on d4T-based therapy.

## Patients and Methods

### Population Studied

All patients and controls were recruited at the same HIV-1 infection clinic at the *Hospital de la Santa Creu i Sant Pau*, which attended a population of 3355 HIV-1-infected patients between 1994 and 2010. All were patients on active follow up with an established diagnosis of HIV-1 infection on d4T-based antiretroviral therapy. Patients were eligible if they had developed pancreatitis and/or peripheral neuropathy while receiving therapy with d4T as part of their antiretroviral regimes. Controls were subjects followed in the same cohort who did not develop pancreatitis and/or neuropathy while on d4T therapy. Controls were selected in a 3∶1 ratio with respect to cases. They were matched to patients with respect to age (±5 years), sex, and exposure to d4T (but not time on d4T). A normal dose of d4T was 40 mg twice daily if the patient weighed >60 kg, and 30 mg twice daily if the patient weighed <60 kg, whereas a reduced dose was 30 mg/12 h and 20 mg/12 h for patients with a weight of ≥60 or <60 kg, respectively. Subjects who were hospitalized or had a frank cognitive impairment such as delirium or dementia on enrolment were not eligible. Patients with opportunistic infections, neoplasms or fever of undetermined origin were excluded from the study too. Written informed consent was obtained from the patients at study entry. The study was approved by the Ethics Committee of the *Hospital de la Santa Creu i Sant Pau*.

### Definition of Clinical Endpoints

A case of acute pancreatitis was defined as a clinical history consistent with pancreatitis (i.e. abdominal pain with or without findings of shock and hypotension where other causes of abdominal pain have been excluded) with supporting biochemical evidence of pancreatitis: elevated lipase (3 times the upper normal limit) or amylase (3 times the upper normal limit) and evidence of pancreatitis from radiological investigation or hemorrhagic pancreatitis at laparotomy or post-mortem exam [9], [10]. Asymptomatic elevations of pancreatic enzymes were not considered to be cases of acute pancreatitis. Chronic pancreatitis cases were excluded from the study.

The diagnosis of neuropathy (sensory or mixed) required the presence of numbness, paresthesias, or dysesthesias in the patient’s lower or lower and upper extremities with onset after starting a d4T-based antiretroviral regime [11], [12]. A confirmatory electromyogram or a nerve conduction analysis was not a compulsory criterion for the diagnosis of neuropathy. The diagnosis of AIDS was based on the 1993 revised case definition of the Centers for Disease Control and Prevention [13].

The presence or absence of lipoatrophy, lipohypertrophy, and mixed syndrome was determined as previously described [7]. The metabolic syndrome was defined according to the U.S. National Cholesterol Education Program (NCEP) Adult Treatment Panel III Guidelines [14] and modified as recommended in the latest American Heart Association/National Heart, Lung, and Blood Institute Scientific Statement [15]. Alcohol abuse was defined when alcohol intake was >40 g daily [16].

### Biochemistry Laboratory Measurements

All laboratory investigations were performed after a 12 h overnight fast and at least 15 minutes after the placement of a peripheral intravenous catheter, as previously described [7], [8].

### Genotyping Analyses

The genomic DNA was extracted from the peripheral leucocytes by the salting-out procedure [17]. In the TS gene, the variable number tandem repeat (VNTR) of 28 bp polymorphism and the G→C SNP in the first and second repeat were analyzed. A DNA fragment was amplified using previously described PCR conditions and primers [7], [8], and directly sequenced using an ABI PRISM 3100 Genetic Analyzer (Applied Biosystems, Foster City, CA, USA). This G to C substitution changes a critical residue in the USF E-box consensus element, abolishes USF-1 binding, and alters transcriptional activity. TS genotypes of the patients were classified according to Kawakami and Watanabe into two groups: high expression type (2/3G, 3C/3G and 3G/3G) and low expression type (2/2, 2/3C and 3C/3C) [18].

The MTHFR gene polymorphisms (677C→T [rs1801133] and 1298 A→C [rs1801131) were determined. These two polymorphisms were analyzed using Fluidigm’s Biomark system. This technology is designed for the allelic discrimination 5′ nuclease assay. The samples and the TaqMan Gene expression assays (Applied Biosystems, Foster City, CA, USA) were prepared following manufacturer’s instructions. The 48.48 dynamic arrays used were automatically loaded using an IFC Controller (Fluidigm Corporation), and real-time reactions were performed and analyzed using BioMark Real-Time PCR System and Analysis software (Fluidigm Corporation), respectively. As a quality control, normal, heterozygote and homozygote sequenced samples were included on every array for each genotype. MTHFR genotypes were classified also into two groups: those associated with a decreased enzymatic activity (homozygous 677T, homozygous 1298C and compound heterozygous patients), and genotypes associated with an increased enzymatic acitivity (heterozygous and wild-type patients) [19]. Combined TS and MTHFR genotypes with respect to enzyme activities produced four groups: low TS expression and increased MTHFR activity (B+A), ow TS expression and decreased MTHFR activity (B+B), high TS expression and increased MTHFR activity (A+A), and high TS expression and decreased MTHFR activity (A+B).

### Statistical Analyses

Data are expressed as median with interquartile range (IQR) or as otherwise specified. Continuous variables were assessed with the nonparametric Mann-Whitney test and categorical data such as genotype and allele frequencies were compared by use of the Fisher’s exact test. The level of significance was established at the 0.05 level and all reported P values are two-sided. A logistic regression analysis was used to examine the association of peripheral neuropathy and/or pancreatitis with TS and MTHFR polymorphisms and other parameters; variables associated with a P<0.1 in the univariate analyses were included in the multivariate stepwise analysis. All analyses were performed with the SAS version 9.1.3 software (SAS Institute Inc., Cary, NC).

## Results

### Population Studied

From January 1994 to December 2010, there were 102 cases of pancreatitis and/or peripheral neuropathy in our cohort**;** 34 cases of pancreatitis, 62 peripheral neuropathies and 6 cases with both pancreatitis and neuropathy. Of them, 63 (61.7%) were associated with d4T exposure, which had been taken at any time by 899 patients for a total exposure of 39,684 months. The incidence of d4T-associated pancreatitis and/or neuropathy was 19.05 (95% CI: 14.28–24.39) cases per 1000 patient-years (PY) in those exposed to d4T and 4.28 (3.13–5.86) in those not exposed (P<0.0001). For peripheral neuropathy the incidences were 18.75 (14.62–24.05) and 1.98 (1.25–3.14), respectively (P<0.0001), whereas for pancreatitis they were 10.28 (7.35–14.39) and 2.3 (1.50–3.54), respectively (P<0.0001). We studied 172 HIV-1-infected, Caucasian patients, exposed to a d4T-based therapy (43 cases and 129 controls). There were no differences between the 43 cases studied and the 20 patients excluded because of lack of DNA. Demographic, means of acquiring HIV-1 infection and the immunovirological status of patients and controls are shown in table 1. There were 127 men (73.8%) and 45 women (26.2%), with a mean age of 51.1±10.9 years (median: 49.0 [IQR: 42.0–58.0] years). Sixty-nine patients (40.1%) had had a prior AIDS-defining condition. Eight patients (18.6%) had pancreatitis, and 35 (81.4%) had peripheral neuropathy. Prior AIDS was significantly more frequent in cases than in controls (OR = 2.36; 95%CI: 1.10–5.07, P = 0.0247). No patient was diabetic or was using insulin or hypoglycemic agents. Alcohol abuse was documented in 11 patients (6.3%). The median daily alcohol consumption for alcohol abusers was 63.0 (IQR: 55.0–94.5) grams, without statistically significant differences between cases and controls (P = 0.8738).

**Table 1:** Demographics and immunovirological status of the population studied.

	Cases (N = 43)	Controls (N = 129)	P value
**Age (yrs.)**	48.0 (43.0–57.7)	50.0 (42.0–58.2)	0.9042
**Males, n (%)**	32 (74.4)	95 (73.6)	0.9999
**Risk group**			
**MsM, n (%)**	17 (39.5)	45 (34.9)	0.3366
**Htsx, n (%)**	14 (32.5)	55 (42.6)	
**IDU, n (%)**	11 (23.2)	30 (21.7)	
**Other** * **, n (%)**	2 (4.6)	1 0.8)	
**Years since diagnosis**	13.0 (9.0–15.7)	13.0 (10.0–17.0)	0.4065
**AIDS, n (%)**	24 (55.8)	45 (34.9)	0.0195
**Smokers, n (%)**	17 (39.5)	70 (54.3)	0.1138
**Alcohol abuse, n (%)**	1 (2.3)	10 (7.7)	0.2952
**HBV co-infection, n (%)**	6 (13.9)	11 (8.5)	0.3751
**HCV co-infection, n (%)**	13 (30.2)	40 (31.0)	0.9999
**Current CD4 count, cells/mm^3^**	515 (346–730)	543 (388–792)	0.5430
**CD4 increase, cells/mm^3^**	386 (194–574)	339 (207–553)	0.6044
**Current CD8 count cells/mm^3^**	834 (582–1181)	874 (617–1146)	0.6521
**CD8 increase, cells/mm^3^**	555 (144–799)	370 (122–594)	0.1180
**Nadir CD4 cell count, cells/mm^3^**	83 (23–206)	182 (54–314)	0.0091
**Nadir CD4 cell count <100 cells/mm^3^, n (%)**	24 (55.8)	45 (35.1)	0.0201
**Nadir CD4 cell count <200 cells/mm^3,^ n (%)**	32 (74.4)	71 (55.5)	0.0314
**Viral load, log_10_, copies/ml**	1.28 (1.28–1.28)	1.28 (1.28–1.55)	0.8307
**Undetectable viral load, n (%)**	33 (76.7)	91 (70.5)	0.5564
**Maximum viral load, log_10_, copies/ml**	5.46 (4.70–5.79)	5.19 (4.29–5.58)	0.1479
**Maximum viral load ≥5 log_10_, copies/ml**	31 (68.9)	75 (55.6)	0.0643
**Viral load decrease, log_10_, copies/ml**	3.78 (2.84–4.28)	3.58 (2.53–4.16)	0.3725

Values are expressed as median and interquartile range, unless indicated.

* includes 2 patients with post-transfusion HIV and 1 with unknown risk, MsM = men who have sex with men, HTSX = heterosexuals, IDU = intravenous drug users, AIDS = acquired immune deficiency syndrome, HCV = hepatitis C virus, HBV = hepatitis B virus, ml = milliliters.

### Antiretroviral Drug Exposure and Immunovirological Status

Most of the patients (124, 72.9%) had undetectable viral load at the time of the study. The median viral load for those who had it detectable was 2.0 (IQR: 1.68–3.06) log_10_ copies/ml. The mean CD4 count was 589±291 cells/mm^3^ (median: 539 [IQR: 383–784]). Nadir CD4 cell count was <200 cells/mm^3^ in 109 patients (60.5%) and <100 cells/mm^3^ in 75 (41.7%). A CD4 cell count nadir <200 cells/mm^3^ was significantly more frequent in cases (OR = 2.34; 95%CI: 1.03–5.58, P = 0.0437) (Table 1). The maximum viral load was above 5 log_10_ copies/ml in 106 patients (61.6%). Four cases (9.3%) and 31 controls (24.0%) were receiving reduced d4T doses (OR = 0.32; 95%CI: 0.08–1.01, P = 0.0630). The cumulated exposure to antiretroviral drugs is shown in table 2. More cases than controls were receiving d4T plus ddI at event (53.5% vs. 34.1%, OR = 2.22; 95%CI: 1.04–4.76, P = 0.0379). None of the patients was receiving hydroxyurea.

**Table 2:** Antiretroviral drug exposure in the population studied.

Parameter	Cases (n = 43)	Controls (n = 129)	P value
ART composition at event			0.0154
**PI-based, n (%)**	33 (76.7)	69 (53.5)	
**NNRTI-based, n (%)**	10 (23.2)	51 (39.5)	
**3 NRTIs, n (%)**	0 (0.0)	9 (7.0)	
NRTI backbone at event			
**d4T+3TC, n (%)**	15 (34.9)	69 (53.5)	0.3335
**d4T+ddI, n (%)**	23 (53.5)	44 (34.1)	
**d4T+ABC, n (%)**	2 (4.6)	6 (4.6)	
**d4T alone, n (%)**	2 (4.6)	6 (4.6)	
**d4T+3TC+ABC, n (%)**	1 (2.3)	3 (2.3)	
**d4T+3TC+ddI, n (%)**	0 (0.0)	1 (0.8)	
Current d4T use, n (%)	0(0)	29 (22.5)	0.0015
Current AZT use, n (%)	13 (30.2)	4 (3.1)	<0.0001
ART duration (m)	110.0 (93.0–138.7)	115.0 (94.7–143.2)	0.4783
Individual drug exposure			
**AZT exposure, m**	43.0 (2.0–70.7)	11.0 (0.0–41.0)	0.0013
**AZT exposure, g**	559.6 (21.2–998.2)	158.8 (0.0–583.8)	0.0026
**d4T exposure, m**	26.0 (9.0–47.7)	57.0 (38.7–76.0)	<0.0001
**d4T exposure, g**	62.9 (21.8–109.8)	135.5 (86.7–177.2)	<0.0001
**d4T exposure, mg/kg**	1.15 (1.04–1.27)	1.06 (0.92–1.18)	0.0126
**3TC/FTC exposure, m**	68.0 (38.0–91.7)	70.0 (36.0–103.2)	0.6790
**ddI exposure, m**	11.0 (3.2–33.0)	17.0 (0.0–60.2)	0.2688
**ddC exposure, m**	0.0 (0.0–0.0)	0.0 (0.0–0.0)	0.7167
**ABC exposure, m**	2.0 (0.0–46.2)	0.0 (0.0–26.7)	0.0406
**TDF exposure, m**	0.0 (0.0–45.0)	14.0 (0.0–43.0)	0.3340
**EFV exposure, m**	0.0 (0.0–0.0)	2.0 (0.0–63.0)	0.0947
**NVP exposure. m**	6.0 (0.0–46.0)	2.0 (0.0–50.2)	0.6660
**ETV exposure, m**	0.0 (0.0–0.0)	0.0 (0.0–0.0)	0.3068
**PI exposure, m**	109.0 (28.2–168.7)	46.0 (22.7–97.5)	0.0123
**NRTI exposure, m**	196.0 (157.2–247.5)	224.0 (172.0–267.2)	0.0819

All parameters expressed as median and (interquartile range) unless indicated. HALS = HIV-1/HAART-associated lipodystrophy syndrome, ART = antiretroviral therapy, PI = protease inhibitor, NNRTI = non-nucleoside reverse transcriptase inhibitor, NRTI = nucleoside reverse transcriptase inhibitor, d4T = stavudine, 3TC = lamivudine, FTC = emtricitabine, TDF = tenofovir, ddI = didanosine, AZT = zidovudine, ddC = zalcitabine, ABC = abacavir, EFV = efavirenz, NVP = nevirapine, ETV = etravirine, m = months, g = grams, kg = kilograms.

### Metabolic Data, Metabolic Syndrome and HALS

Metabolic and fat data are shown in table 3. There were no differences between cases and controls with respect to anthropometric and metabolic parameters. Sixty-nine patients (38.3%) had metabolic syndrome, without differences between cases and controls (OR = 1.59; 0.75–3.32, P = 0.2498). Ninety-three patients (54.1%) had HALS. HALS was less frequent in cases than in controls (41.9% vs. 58.1%, OR = 0.52; 95%CI: 0.24–1.10, P = 0.0932).

**Table 3:** Anthropometric, metabolic and fat data in the population studied.

	Cases (n = 43)	Controls (n = 129)	P value
**Weight, kg**	64.7 (60.0–73.7)	65.5 (58.0–73.0)	**0.7542**
**BMI, kg/m^2^**	23.5 (20.5–25.8)	23.8 (21.3–25.7)	**0.5769**
**Waist circumference, cm**	89.0 (81.2–93.7)	89.0 (82.7–95.0)	**0.8006**
**WHR**	0.96 (0.90–1.02)	0.95 (0.90–1.02)	**0.8987**
**LSGS score, units**	4.5 (2.0–10.0)	7.0 (3.4–11.1)	**0.0386**
**Facial score, units**	1.0 (0.0–2.0)	2.0 (0.7–2.0)	**0.0222**
**Systolic BP, mm Hg**	120 (120–138)	120 (110–130)	**0.2025**
**Diastolic BP, mm Hg**	75 (70–80)	75 (70–80)	**0.6513**
**Metabolic syndrome, n (%)**	21 (46.7)	48 (35.5)	**0.2498**
**Total cholesterol, mmol/l**	5.07 (4.18–6.03)	5.08 (4.44–5.92)	**0.8966**
**Triglycerides, mmol/l**	1.90 (1.17–3.22)	1.63 (1.12–2.54)	**0.2752**
**HDL cholesterol, mmol/l**	1.11 (0.95–1.32)	1.23 (0.97–1.53)	**0.1690**
**LDL cholesterol, mmol/l**	2.89 (2.28–3.65)	2.89 (2.46–3.50)	**0.6693**
**VLDL cholesterol, mmol/l**	0.88 (0.54–1.19)	0.77 (0.52–1.11)	**0.3723**
**Fasting glucose, mmol/l**	5.30 (5.02–5.87)	5.30 (4.90–6.00)	**0.8317**
**HALS, n (%)**	18 (41.9)	75 (58.1)	**0.0932**

All values expressed as median and (interquartile range) unless specified. HALS = HIV-1/HAART-associated lipodystrophy syndrome, BMI = body mass index, WHR = waist-hip ratio, LSGS = lipodystrophy severity grade score, mmol/l = milimoles per liter, HDL = high density lipoprotein, LDL = low density lipoprotein, VLDL = very low density lipoprotein, pmol/l = picomoles per liter, g = grams.

### TS and MTHFR Polymorphisms, and Development of Pancreatitis and/or Peripheral Neuropathy

The distribution of the different genotypes is shown in table 4. Ninety-one patients (52.9%) had a low expression TS genotype. This genotype was more frequent in patients with pancreatitis and/or peripheral neuropathy than in controls (72.1% vs. 46.5%, OR = 2.97; 95%CI: 1.33–6.90, P = 0.0062). The association remained statistically significant when peripheral neuropathy alone was considered (68.6% vs. 46.5%, OR = 2.51; 95%CI: 1.07–6.14, P = 0.0336), whereas there was a trend to significance for pancreatitis (87.5% vs. 46.5%, OR = 8.05; 95%CI: 0.98–367.94, P = 0.0592). Neither 677C→T (P = 0.1119) or 1298A→C (P = 0.0708) MTHFR polymorphisms were associated with the development of pancreatitis and/or peripheral neuropathy. However, when functional translation was taken into account MTHFR genotypes associated with increased enzymatic activity were more frequent in cases than in controls (79.1% vs. 56.6%, OR = 2.90, 95%CI: 1.23–7.41, P = 0.01423). The association remained statistically significant for peripheral neuropathy (77.1% vs. 58.4%, OR = 2.40, 95%CI: 0.97–6.55, P = 0.0417), but not for pancreatitis (87.5% vs. 60.9%, OR = 4.48, 95%CI: 0.55–205.51, P = 0.2553).

**Table 4:** Gene polymorphisms associated with the presence of pancreatitis and/or neuropathy in d4T-exposed patients.

	Cases (n = 43)	Controls (n = 129)	P value
**TS genotype**			
**2R/2R, n (%)**	9 (20.9)	19 (13.9)	**0.0618**
**2R/3C, n (%)**	15 (34.9)	30 (23.2)	
**2R/3G, n (%)**	7 (16.3)	35 (27.1)	
**3C/3C, n (%)**	7 (16.3)	11 (8.5)	
**3C/3G, n (%)**	5 (11.6)	25 (19.4)	
**3G/3G, n (%)**	0 (0.0)	10 (7.7)	
**Low expression, n (%)**	31 (72.1)	60 (46.5)	**0.0046**
**High expression, n (%)**	12 (27.9)	69 (53.5)	
**MTHFR 677C→T genotype**			
**C/C, n (%)**	21 (48.8)	42 (32.5)	**0.1119**
**C/T, n (%)**	18 (41.9)	63 (48.8)	
**T/T, n (%)**	4 (9.3)	24 (18.6)	
**MTHFR 1298A→C genotype**			
**A/A, n (%)**	22 (51.2)	64 (49.6)	**0.0708**
**A/C, n (%)**	14 (32.5)	58 (44.9)	
**C/C, n (%)**	7 (16.3)	7 (5.4)	
**MTHFR genotypes and enzymatic activity**			
**Genotypes with increased activity** * **, n (%)**	34 (79.1)	73 (56.6)	**0.0066**
**Genotypes with decreased activity^†^, n (%)**	9 (20.9)	56 (20.9)	
**Combined TS and MTHFR genotypes**			
**Low expression and increased activity (B+A), n (%)**	25 (58.1)	34 (26.3)	**0.0013**
**Low expression and decreased activity (B+B), n (%)**	6 (13.9)	26 (20.1)	
**High expression and increased activity (A+A), n (%)**	9 (20.9)	39 (30.2)	
**High expression and decreased activity (A+B), n (%)**	3 (6.9)	30 (23.2)	

TS = thymidylate synthase, MTHFR = methylene-tetrahydrofolate reductase,

* Heterozygous and wild-type patients, ^†^Homozygous 677T, homozygous 1298C and compound heterozygous patients.

Combined TS and MTHFR genotypes with respect to peripheral neuropathy or pancreatitis are shown in table 4, where it is apparent that those combinations with a greater negative impact on TS activity were more frequently seen in cases.

### Factors Associated with Development of Pancreatitis and/or Peripheral Neuropathy

A multivariable analysis was performed taking as the dependent variable the development of pancreatitis and/or peripheral neuropathy and as independent variables, age, sex, AIDS, CD4 count nadir <200/mm^3^, CD4 nadir <100/mm^3^, d4T exposure (m), d4T exposure (g), d4T exposure (mg/kg), AZT exposure (m), AZT exposure (g), ABC exposure (m), d4T plus ddI at event, EFV exposure (m), PI exposure (m), NRTI exposure (m), HALS, TS genotype (low vs. high expression),MTHFR genotype (increased vs. decreased enzymatic activity), and combined TS and MTHFR genotypes, all variables associated with a P value <0.1 in the univariate analysis. Independent positive or negative predictors for the development of d4T-associated pancreatitis and/or peripheral neuropathy were: combined TS and MTHFR genotypes (reference: A+A; P = 0.002; OR_A+B_ = 0.34 [95%CI: 0.08 to 1.44], OR_B+A_ = 3.38 [95%CI: 1.33 to 8.57], OR_B+B_ = 1.13 [95%CI: 0.34 to 3.71]), nadir CD4 cell count >200 cells/mm^3^ (OR = 0.38; 95%CI: 0.17–0.86, P = 0.021), and HALS (OR = 0.39 95%CI: 0.18–0.85, P = 0.018).

## Discussion

Our study suggests an association between TS and MTHFR polymorphisms and the appearance of d4T-related toxicity in the form of acute pancreatitis or peripheral neuropathy. However, our work has inherent limitations. First, this is a case-control study and therefore no causal relationships should or must be drawn. Second, in case-control studies endpoint verification is of paramount importance. It is known that peripheral neuropathy or acute pancreatitis in the setting of HIV-1 infection treated with HAART may be caused not only by antiretroviral drugs but also by other drugs, alcohol abuse, or biliary stones and hypertriglyceridemia in the case of pancreatitis [20]. On the other hand, there is no pathognomic test to etiologically ascribe the etiology of peripheral neuropathy or pancreatitis. Notwithstanding that, none of these well-known causes was present when patients in the study were diagnosed as having peripheral neuropathy or pancreatitis, except for alcohol abuse which in fact was more frequent among controls. Unfortunately, when the study was performed, intracellular d4T-TP concentrations in PBMCs were not available. Third, d4T is a well-known cause of peripheral neuropathy and pancreatitis, especially when combined with didanosine (ddI) or even more when combined with ddI and hydroxyurea [21]. Among our cases, 23 (53.5%) were also exposed to ddI, which may have contributed to the development of pancreatitis and/or peripheral neuropathy. Although cumulated exposure to ddI was not different in cases and controls, taking d4T plus ddI at event was statistically more frequent in cases. However, taking both drugs in combination was not an independent predictor for the development of peripheral neuropathy and/or acute pancreatitis. Fourth, there are a number of gene markers which have been associated with the risk of developing both toxic peripheral neuropathy and pancreatitis in HIV-infected patients on HAART, such as cystic fibrosis trans-membrane conductance regulator (CTR) and serine protease inhibitor kazal-1 (SPINK-1) mutations for pancreatitis and mitochondrial haplotype T for peripheral neuropathy [22]–[24]. We cannot exclude the possibility that some of these untested genes may have contributed to acute pancreatitis and/or peripheral neuropathy in our patients.

Acute pancreatitis in the setting of HIV infection and antiretroviral therapy has wide incidence rates ranging from 1.27 to 22.6events/1000 PY [25]–[28]. These wide incidence rates may be explained by different diagnostic criteria (clinical vs. laboratory-based), and by the fact that some of the studies were performed in early calendar years, when more toxic drugs were used. Its appearance has been linked to the classical risk factors; i.e. alcohol abuse, hypertriglyceridemia, as well as to the use of antiretroviral drugs such as d4T and ddI [25]–[27]. The incidence rates for peripheral neuropathy have also been very wide, ranging from 0.7 to 39.7/1000 PY [29]. Known risk factors for developing peripheral neuropathy in HAART-treated patients include alcohol abuse, treatment with other neuropathic drugs (isoniazid, methotrexate,…), factors which were ruled out in our patients [30]. However, sometimes it is difficult to distinguish toxic peripheral neuropathy from exacerbation of HIV-associated neuropathy in HAART-treated patients, which may also contribute to these varying incidence rates. Notwithstanding that, none of our patients had clinical features consistent with peripheral neuropathy prior to taking d4T. The incidence of both acute pancreatitis and peripheral neuropathy in our work fall within these wide ranges, most probably because our study period spans throughout a long time period when mitochondrially-toxic drugs were still widely used.

Acute pancreatitis has been associated with mutations in CTR and SPINK-1 genes both in the general population and in HIV-infected patients [22], [23]. Among HIV-infected patients with acute clinical pancreatitis in a small study, 40% were carriers of CTR or SPINK-1 mutations [31]. Similarly, a number of gene polymorphisms have been described, associated with an increased risk of developing peripheral neuropathy associated with d4T and ddI use, including mitochondrial haplotype T [32], the mitochondrial polymorphism MTND2/HON4917G, specific to haplogroup G [32], and the 282 C>Y hemochromatosis gene mutation [33].

Among the factors independently associated with the development of neuropathy or pancreatitis, we found a low CD4 cell count nadir, a common marker for most toxicities and co-morbidities [34], [35]. Most probably it is a signal of increased inflammation and/or immune activation, which in turn contribute to overexpression of nucleoside transporters potentially leading to increased intracellular d4T-TP levels [36]. The association of TS and MTHFR polymorphisms with mitochondrial toxicity has biological plausibility, since genotypes which have a negative impact on TS activity are associated with higher d4T-TP intracellular levels and with fat redistribution, a well-known toxicity of mitochondrial origin [7], [8]. Not surprisingly, we found that low expression TS genotypes are also associated with the appearance of d4T-induced mitochondrial toxicity in the form of peripheral neuropathy whereas the association for acute pancreatitis showed only a trend to statistical significance, most likely because of the small number of cases. A potentially contributing factor could have been that cases had a higher per weight exposure to d4T than controls, but again this was not an independent predictor of development of neuropathy or acute pancreatitis.

MTHFR gene is also polymorphic. The best known polymorphism consists of a 677 C**→**T transition in exon 4, which results in an alanine to valine substitution in the predicted catalytic domain of MTHFR. This substitution renders the enzyme thermolabile and homozygotes and heterozygotes have about 70% and 35% reduced enzyme activity, respectively [37]. A second common polymorphism in the MTHFR gene is a 1298 A**→**C transition in exon 7 which results in a glutamate to alanine substitution within a presumed regulatory domain of the protein [38]. The 1298C allele leads to a decreased enzyme activity, and individuals who are compound heterozygous for the 677T and 1298C have a 40–50% reduced MTHFR activity [19]. Therefore, the non-mutated forms of MTHFR exhibit high enzymatic activity and should lead to high levels of intracellular 5,10-methylene tetrahydrofolate that will favor the formation and stability of the inhibitory ternary complex involving TS, 5,10-methylene tetrahydrofolate and deoxyuridine monophosphate [19]. Consequently, patients with non-mutated alleles should have a decreased TS activity, which in turn may lead to increased d4T-TP intracellular levels and to clinical toxicity including pancreatitis and peripheral neuropathy.

Stavudine-associated side effects may seem of only limited relevance today, because its use has greatly decreased in developed countries mainly due to its association with fat distribution abnormalities [39]. However, its use in developing countries as part of the starting antiretroviral regimes, with its associated toll of toxicities [1], is still common despite the WHO recommendation to substitute for less toxic NRTI when feasible [40].

In summary, our study suggests that d4T-associated acute pancreatitis and/or peripheral neuropathy are associated with low-degree TS expression and MTHFR genotype associated with an increased enzymatic activity. There is a plausible pathogenic mechanism for such an association since it is well-known that, in d4T-treated patients, the presence of low-degree TS expression genotype is associated with increased d4T-TP intracellular concentrations and MTHFR genotype associated with an increased enzymatic activity contributes to a decreased TS functionality. This may be of value in tailoring d4T therapy when needed.
